# Bibliometric Analysis of Dental Caries Detection

**DOI:** 10.7759/cureus.40741

**Published:** 2023-06-21

**Authors:** Ganesh C, Victor Samuel A, Deenadayalan Purushothaman, Magesh K T, Vivek N

**Affiliations:** 1 Department of Oral Medicine and Radiology, SRM Kattankulathur Dental College and Hospital, SRM Institute of Science and Technology, Chennai, IND; 2 Department of Pedodontics and Preventive Dentistry, SRM Kattankulathur Dental College and Hospital, SRM Institute of Science and Technology, Chennai, IND; 3 Department of Orthodontics, SRM Kattankulathur Dental College and Hospital, SRM Institute of Science and Technology, Chennai, IND; 4 Department of Oral Pathology, SRM Kattankulathur Dental College and Hospital, SRM Institute of Science and Technology, Chennai, IND; 5 Department of Oral and Maxillofacial Surgery, SRM Kattankulathur Dental College and Hospital, SRM Institute of Science and Technology, Chennai, IND

**Keywords:** dental caries, risk factors, etiology, dentistry, h index

## Abstract

The main purpose of this bibliometric analysis is to conduct a quantitative and qualitative analysis of the publications on dental caries. Research productivity can be measured with the use of bibliometric analysis. In this study, we conducted a bibliometric analysis using the Scopus database to identify dental caries research trends and patterns over the years. The search yielded 1630 scientific articles. The data was analysed using bibliometric indicators such as the h-index, the total number of citations and the number of publications. An analysis of the data highlighted that the United States of America (USA) and Brazil have the highest number of single-country publications. Top authors were listed based on the h-index and the total number of citations. Top cited countries, institutions etc. were also analysed using this bibliometric study. This bibliometric evaluation provides a wide area of literature carried out to date. The existing knowledge can be used to direct future research.

## Introduction and background

Dental caries, also known as cavities or tooth decay, is a significant public health issue affecting individuals of all ages worldwide. Despite the widespread availability of preventive measures, the prevalence of dental caries remains high in many populations [1]. To address this challenge, a current understanding of the dental caries knowledge that exists, including the risk factors, preventive strategies, and management is paramount. Bibliometrics is a method for quantitatively analysing the literature in a specific field, such as dental caries, and providing valuable insights into the distribution, frequency, and patterns of research activities. Bibliometric studies can help identify the most productive authors, institutions and countries, as well as reveal trends and gaps in the research landscape.

This bibliometric analysis of the scientific literature on dental caries aimed to provide a comprehensive overview of the current knowledge and research trends in this field. The study encompassed an extensive literature search and analysis of relevant studies published in the past decade that covered various aspects of dental caries including its aetiology, risk factors, prevention and management. The findings of this bibliometric analysis may provide valuable insights into the current state of knowledge about dental caries including critical public health issues and highlight areas for future research. The findings of the study may be used as a valuable resource for researchers, practitioners, and policy-makers in dental health.

## Review

Methodology

Bibliometric analysis is a valuable tool for identifying trends and patterns in research. Briefly, it involved the quantitative analysis of publication data. Insights, such as productivity, impact, and the collaborative networks of researchers and institutions were extracted. In this study, the Scopus database was used to identify the trends and patterns in dental caries research over the years.

Data extraction

We conducted a comprehensive search of the Scopus database using relevant keywords, such as "dental caries, tooth decay", “dental caries epidemiology", "dental caries prevention" and "dental caries treatment." The search was limited to published articles, reviews, and conference papers. The search yielded 1630 scientific articles.

Data analysis

The data was analysed using bibliometric indicators, such as the h-index, the total number of citations and the number of publications. The data was also visualised using VOSviewer and Biblioshiny, with the help of R-studio software (R Foundation for Statistical Computing, Vienna, Austria).

Results

Most Cited Local Documents

The identified articles were analysed to determine the top eight locally cited articles. The top eight articles are listed below (Table 1). According to the citation criteria, Lussi (1999) [2] has the highest number of citations compared to the other articles. Lussi, published in the journal Caries Research, has been cited approximately 115 times (Table 1).

**Table 1:** Most locally cited documents

Authors	Citations	Year	Title	Journal
Lussi A [2]	115	1999	Performance and reproducibility of a laser fluorescence system for detection of occlusal caries in vitro.	Caries Research
Lussi A [3]	95	2001	Clinical performance of a laser fluorescence device for detection of occlusal caries lesions.	European Journal of Oral Sciences
Bader JD [4]	89	2004	A systematic review of the performance of a laser fluorescence device for detecting caries.	The Journal of the American Dental Association
Jablonski-Momeni A [5]	86	2008	Reproducibility and accuracy of the ICDAS-II for detection of occlusal caries in vitro.	Caries Research
Shi X-Q [6]	81	2000	Occlusal caries detection with KaVo DIAGNOdent and radiography: an in vitro comparison.	Caries Research
Ekstrand KR [7]	71	2007	Detection and activity assessment of primary coronal caries lesions: a methodologic study.	Operative Dentistry
Lussi A [8]	60	2004	DIAGNOdent: an optical method for caries detection.	Journal of Dental Research
Ekstrand KR [9]	60	1998	Detection, diagnosing, monitoring and logical treatment of occlusal caries in relation to lesion activity and severity: an in vivo examination with histological validation.	Caries Research

Most Cited Global Documents

Based on the global citation criteria, the identified articles were analysed to determine the top eight global-cited articles. The result highlighted that Ismail (2007) [10], published in Community Dentistry and Oral Epidemiology, has the highest number of citations, with approximately 831 citations (Table 2).

**Table 2:** Most globally cited documents

Author	Citations	Year	Title	Journal
Ismail AI [10]	831	2007	The international caries detection and assessment system (ICDAS): an integrated system for measuring dental caries.	Community Dentistry and Oral Epidemiology
Pinheiro ET [11]	375	2003	Microorganisms from canals of root-filled teeth with periapical lesions.	International Endodontic Journal
Lussi A [2]	349	1999	Performance and reproducibility of a laser fluorescence system for detection of occlusal caries in vitro.	Caries Research
Pitts N [12]	315	2004	The dental caries experience of 14-year-old children in England and Wales. Surveys co-ordinated by the British Association for the Study of Community Dentistry in 2002/2003.	Community Dental Health
Lussi A [3]	289	2001	Clinical performance of a laser fluorescence device for detection of occlusal caries lesions.	European Journal of Oral Sciences
Lee JH [13]	287	2018	Detection and diagnosis of dental caries using a deep learning-based convolutional neural network algorithm.	Journal of Dentistry
Shi X-Q [6]	261	2000	Occlusal caries detection with KaVo DIAGNOdent and radiography: an in vitro comparison.	Caries Research
Frencken JE [14]	251	2012	Minimal intervention dentistry for managing dental caries – a review: report of a FDI task group.	International Dental Journal

Top Cited Countries

**Figure 1:**
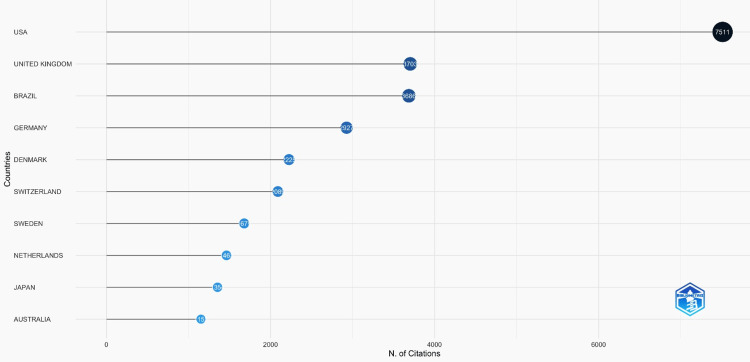
Most cited countries

The top cited articles were analysed using Biblioshiny based on the countries affiliated with the published studies. The United States was involved in the most dental caries research studies compared with the rest of the world, with a massive 7511 citations. This was followed by the United Kingdom with 3703 citations and Brazil with 3686. Australia ranked 10th, with only 15 cited articles (Figure 1).

Institutions With the Most Relevant Articles

**Figure 2:**
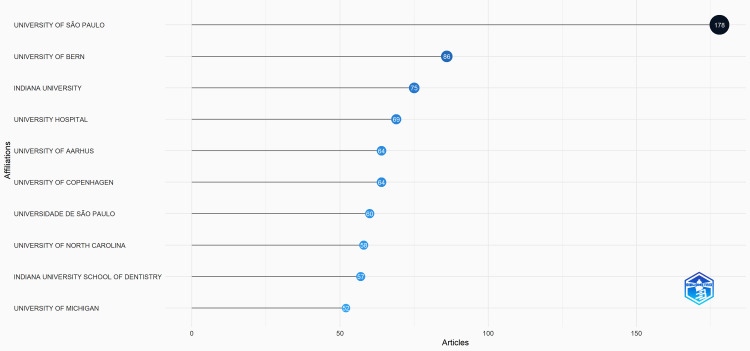
Institutions with the most relevant articles

Based on the bibliometric analysis, the top 10 universities with the most relevant articles on dental caries were listed. The University of São Paulo published the highest number of articles on dental caries (a total of 178 articles), followed by the University of Bern in the second position with 86 articles and Indiana University in the third position with 75 articles (Figure 2).

Keyword Analysis

**Figure 3:**
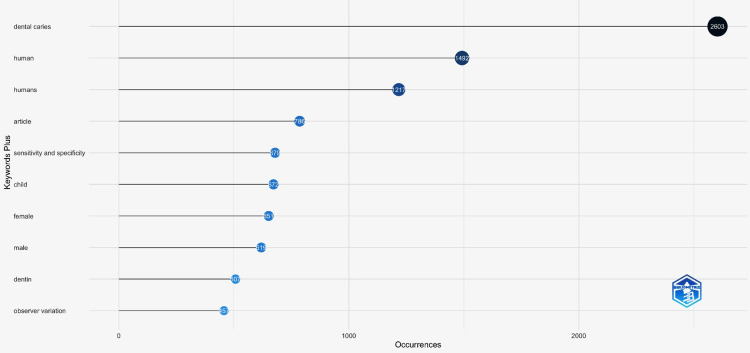
Most relevant keywords

The top 10 most relevant keywords were evaluated. The most frequently used keyword was “Dental caries” with an occurrence of 2603. Other frequently mentioned keywords included “humans” with 1492 occurrences. The other most relevant keywords are listed in Figure 3.

Trending Topics Over Time

**Figure 4:**
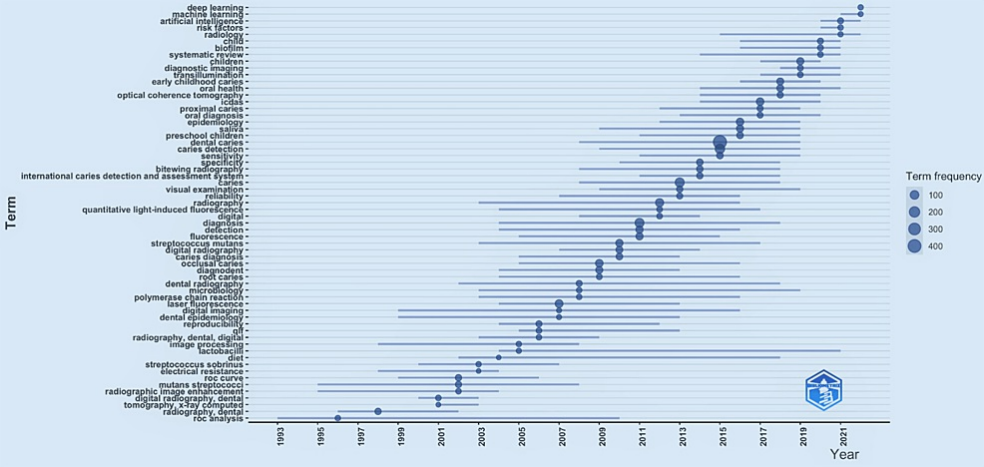
Trending topics over time

From the time research started on the detection of dental caries till the middle of the 1990s, terms such as receiver operating characteristic (ROC) analysis were the most trended topics. In the early 2000s, the most trended topics were “tomography” and “x-ray computer”. After 2020, the top trending topics were “artificial intelligence”, “machine learning” and “deep learning”. These topics are also the presently trending topics related to dental caries. All the trending topics are listed in Figure 4.

Frequency of Words Used Over Time

**Figure 5:**
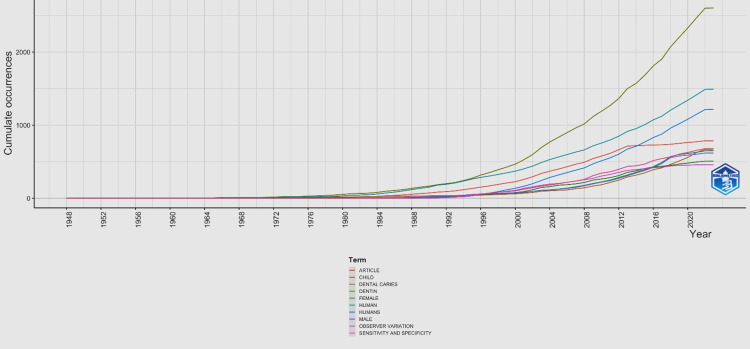
Frequency of words used over time

The top 10 most frequently used words related to dental caries are listed in Figure 5. Most of these words have started to peak. The most frequent terms used after 1988 included “dentin”, which has gradually reached the highest position over recent years, with a cumulative occurrence of over 2000. In recent years after 2020, other words were also mentioned as shown in Figure 5.

Journals Production Over Time

**Figure 6:**
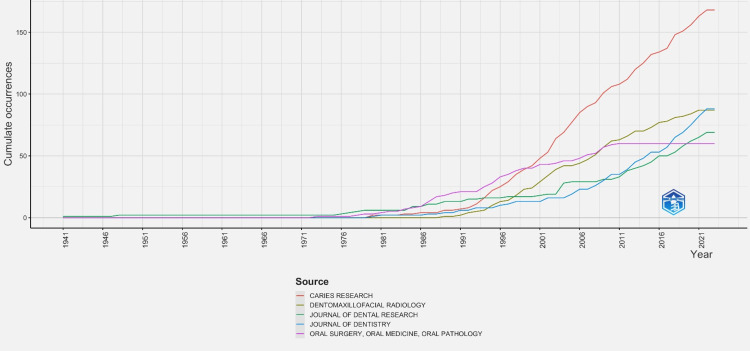
Sources production over time

The top five journals that published dental caries research over time are listed in Figure 6.

On close examination of the analysed data, there were no journals with dental caries publications over time before the year 1975. From 1995 to 2000, there was a close competition between the top five journals. In more recent years, caries research journals have significantly impacted dental caries research, with a massive cumulative occurrence of more than 150 publications.

Annual Scientific Production

**Figure 7:**
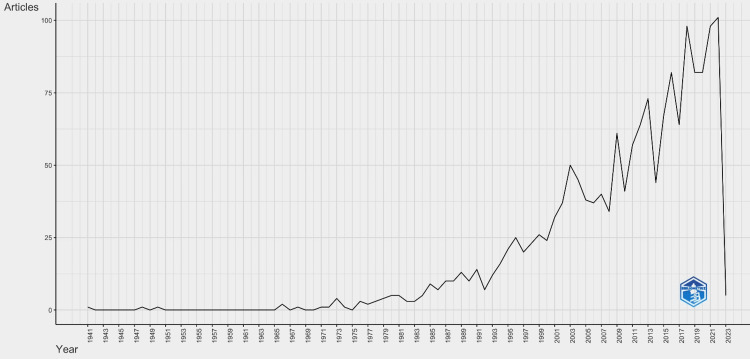
Annual scientific production

On analysing the data over time, the top five highest numbers of articles published in a particular year are listed in Table 3. The highest number of articles published among all journals occurred in 2022, with a massive number of 101 dental caries publications (Figure 7).

**Table 3:** The top five highest numbers of articles published in a particular year

Year	Total no of dental caries articles published in that year
2022	101
2021,2018	98
2020,2019,2016	82
2013	73
2017& 2012	64

H-index

**Figure 8:**
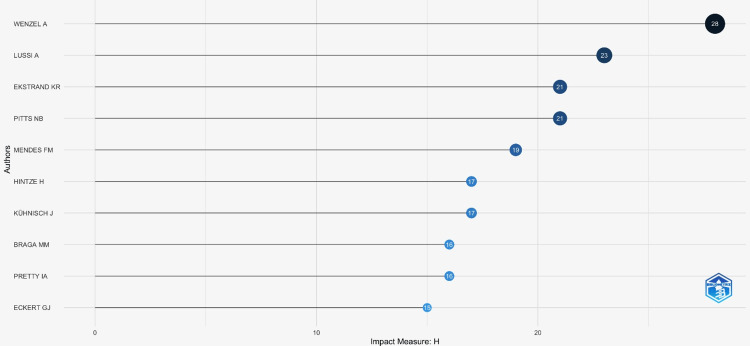
Top 10 authors based on h-index

The h-index, or Hirsch index, measures the impact of a particular scientist rather than a journal. The h-index is “defined as the highest number of publications of a scientist that received h or more citations each while the other publications have not more than h citations each” [15]. Based on the data analysis, the top five authors with the highest h-index were identified (Table 4). The top 10 authors based on the h-index are listed in Figure 8.

**Table 4:** Top five authors with highest h-index

AUTHOR NAME	H-INDEX
WENZEL A	28
LUSSI A	23
EKSTRAND KR	21
PITTS NB	21
MENDES FM	19

Discussion

**Figure 9:**
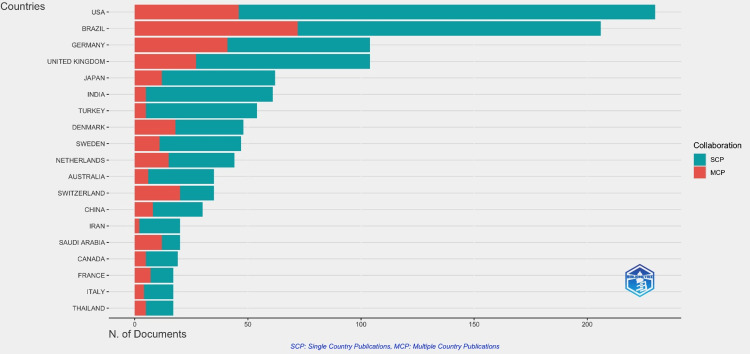
Corresponding authors and their affiliated countries

This bibliometric analysis is the first of its kind. It aimed to identify and quantitatively evaluate scientific research articles on dental caries detection methods. An analysis of the data highlighted that the United States of America and Brazil have the highest number of single-country publications (Figure 9).

The citations are seen in Figure 1. This was attributed to the finding that the majority of publications were from the University of São Paulo in Brazil and Indiana University in the USA (Figure 2).

The two top highly cited authors globally (Table 2), namely, Ismail [10] and Pinheiro [11], were affiliated with the University of Michigan (USA) and the State University of Campinas, Brazil, respectively. Interestingly, the majority of the top 10 journals on dental caries belong to the United Kingdom, the USA, or Switzerland. This indicates that developed countries make significant contributions to this field of research.

Although in recent years, journals such as Caries Research have published more than 150 articles (Figure 6) and these can be correlated with the top eight locally cited publications (Table 1). For example, articles were published by authors, such as Lussi et al. 1999 [2], Jablonski Momeni et al. 2008 [5], and Shi et al. 2000 [6]. Moreover, among the top eight globally cited publications (Table 2), articles included those written by Lussi et al. 1999 [2] and Shi et al. 2000 [6]. Hence, caries research journals significantly contribute to the publication of dental caries research. It was also found that among the top eight globally cited articles (Table 2), Lussi et al. had multiple publications in the years 1999, [2] 2001 [3] and 2004 [8]. The authors were affiliated with the University of Bern, which ranked second among the top 10 universities with most relevant dental caries research articles (Figure 2). This shows that the authors made a significant contribution to their university’s research output. 

**Figure 10:**
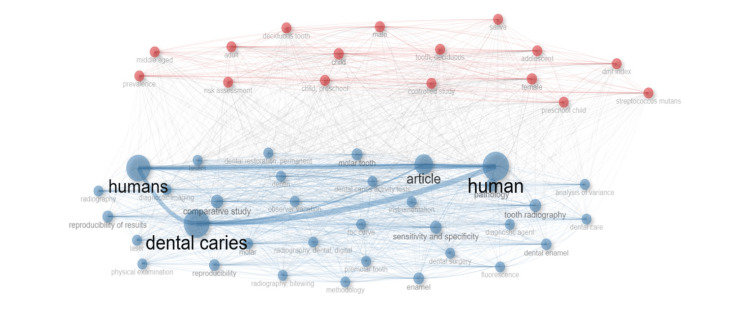
Keywords with the most co-occurrence

Keyword co-occurrence was also analysed. It is said that keywords with the relevance rate are closer the keywords to each other [16]. Our findings determined that the keywords, “dental caries”, “human”, “humans”, and “articles” had the highest co-occurrence (Figure 10).

Co-occurrence of Author Keywords

**Figure 11:**
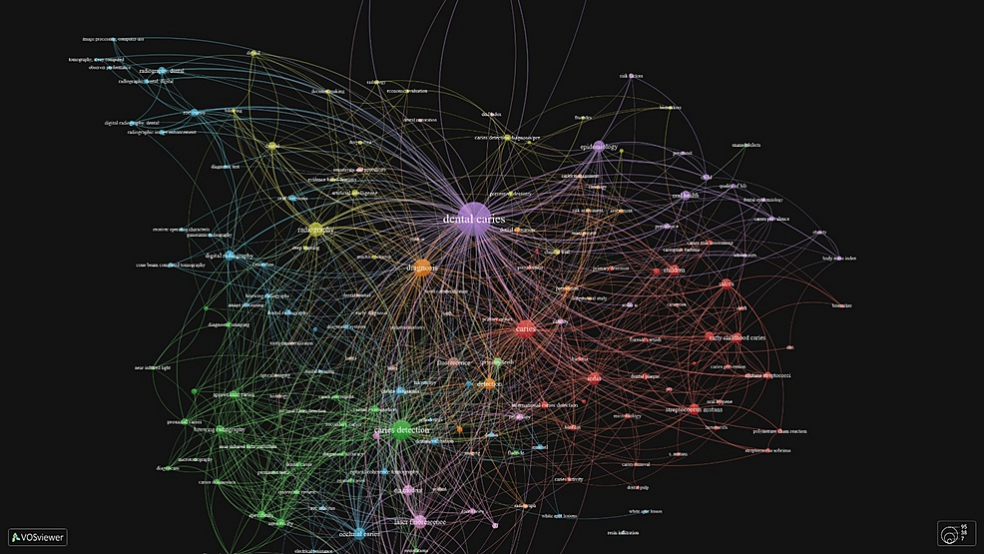
Image obtained from the vos-viewer denotes the co-occurrence of author keywords of different domains

The incidence of co-occurrence of author keywords was investigated and it was considered in terms of the relationship between their research. "DENTAL CARIES”, “CARIES DETECTION”, “CARIES” and “DIAGNOSIS & RADIOGRAPHY" were the most frequently co-occurring terms (Figure 11).

Author Bibliographic Coupling

**Figure 12:**
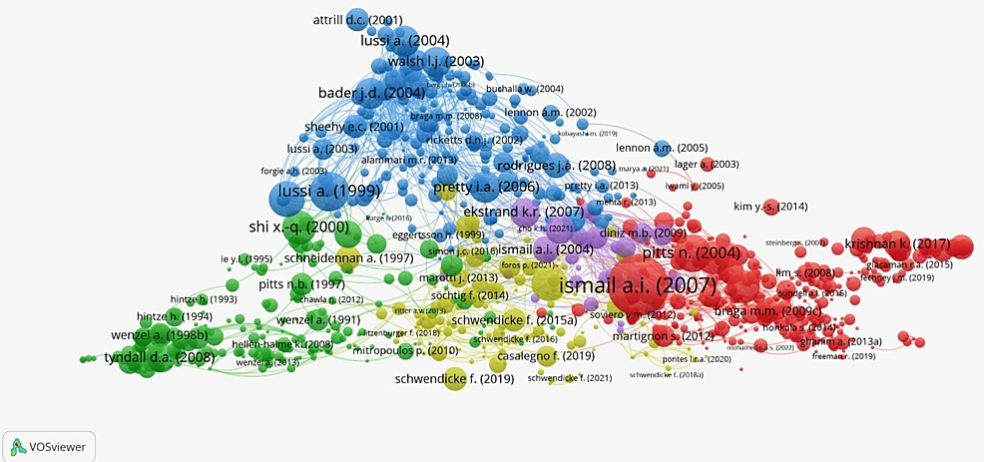
Author Bibliographic Coupling

Author bibliographic coupling is an extension of bibliographic coupling, and refers to the phenomenon when two authors cite the same article(s) in their publications [17]. For example, an article published by Ismail (2007) [10] dominated the author bibliographic coupling, as seen in Figure 12.

Advancements

In recent years, there have been significant advancements in caries treatment through research. These improvements have aimed to provide more effective, minimally invasive, and patient-friendly approaches to managing dental caries. Here are some notable advancements:

Early detection techniques: Research has focused on developing more accurate and efficient methods to detect caries at their earliest stages. Technologies like laser fluorescence, digital imaging, and fluorescence-based diagnostic tools have improved the detection of hidden and early caries lesions, enabling timely intervention.

Remineralization therapies: Researchers have explored various remineralization strategies to reverse early-stage caries. The development of remineralizing agents containing calcium, phosphate, and fluoride ions, as well as bioactive materials like casein phosphopeptide-amorphous calcium phosphate (CPP-ACP), has shown promising results in promoting remineralization and halting the progression of caries.

Minimal intervention techniques: The concept of minimal intervention dentistry has gained traction, aiming to preserve as much healthy tooth structure as possible. Advances in adhesive technologies and dental materials have facilitated minimally invasive approaches such as micro-invasive and non-invasive techniques, including resin infiltration, sealing of occlusal surfaces, and selective removal of carious tissue.

Antibacterial agents: Research has focused on developing effective antibacterial agents that can selectively target cariogenic bacteria while preserving the natural oral microbiota. Innovations include antimicrobial peptides, nanoparticles, and photodynamic therapy, which show promise in reducing cariogenic bacteria and preventing caries progression.

Biomaterials for restoration: Researchers have explored novel biomaterials to improve the longevity and performance of dental restorations. Advances in resin-based composites, glass ionomer cements, and bioactive materials have resulted in improved aesthetic properties, enhanced bonding strength, and increased resistance to wear and secondary caries.

Regenerative approaches: Regenerative dentistry aims to promote the regeneration of damaged dental tissues. Researchers are investigating techniques such as tissue engineering, stem cell therapy, and growth factors to facilitate the regeneration of dentin, enamel, and the pulp-dentin complex, which could potentially lead to non-invasive or less invasive treatments for caries.

Digital dentistry: Technological advancements in digital dentistry have revolutionized caries treatment planning and restoration procedures. Digital imaging, computer-aided design and manufacturing (CAD/CAM), and 3D printing enable precise and efficient restoration fabrication, resulting in better-fitting restorations with reduced chairside time.

These advancements in caries treatment through research offer promising possibilities for improved prevention, detection, and management of dental caries. However, it's important to note that while research is advancing, regular dental check-ups, proper oral hygiene practices, and a healthy diet remain crucial in preventing caries.

Limitations

One of the limitations of this bibliometric analysis is using a single Scopus database. However, the journals in the Scopus database are audited annually to ensure they adhere to higher standards. It is the largest abstract and citation database of peer-reviewed scientific journals, books and conference proceedings. The Scopus database was therefore used in the current study to retrieve data and analyze using software.

## Conclusions

The findings of this review indicated a development in detection of dental caries literature over eight decades. A lack of well-designed clinical research persists involving substances like liquid crystalline systems, polymer-based nanoparticles, lipid-based nanoparticles and inorganic nanoparticles. These lacunae lay the groundwork for future research improvements in the field of dental caries management. 
